# Pregnancy Outcomes among Women with Intermittent Asthma: A Retrospective Cohort Study

**DOI:** 10.3390/ijerph18126376

**Published:** 2021-06-12

**Authors:** Phuttipol Chaiprom, Ratanaporn Sekararithi, Theera Tongsong, Kuntharee Traisrisilp

**Affiliations:** Department of Obstetrics and Gynecology, Faculty of Medicine, Chiang Mai University, Chiang Mai 50200, Thailand; nosphuttipol2525@gmail.com (P.C.); ratanaporn.se@cmu.ac.th (R.S.)

**Keywords:** abortion, asthma, low birth weight, preeclampsia, preterm birth

## Abstract

Background: It is already known that asthma strongly increases risks of poor pregnancy outcomes. We wonder whether intermittent asthma, the least severe form but accounting for the majority of cases, increases such adverse outcomes or not. Therefore, we conducted this study to compare adverse pregnancy outcomes between pregnancies with intermittent asthma and low-risk pregnancies (controls). Methods: The full medical records of pregnancies with intermittent asthma were comprehensively reviewed and low-risk pregnancies were randomly recruited as controls with a ratio of 10:1. The obstetric outcomes were compared between both groups, and the outcomes in the active subgroup of intermittent asthma (defined as at least one asthmatic attack during pregnancy) were also compared with the controls. Results: Of 364 study cases and 3640 controls, the rates of poor outcomes (preterm birth, preeclampsia, fetal growth restriction etc.) were not significantly different. However, cases with active disease slightly, but significantly, increased the risk of low birth weight. Moreover, mean gestational age was significantly lower in the study group. Conclusions: A new insight gained from this study is that intermittent asthma is not associated with poor pregnancy outcomes, but cases with asthmatic attack during pregnancy tended to increase the risk of preterm birth and low birth weight. This information is important for counseling and the planning of antepartum management.

## 1. Introduction

Asthma is one of the most common medical disorders encountered in women of reproductive age. Accumulating data on pregnancies complicated with asthma indicate that asthma is not a benign disease with respect to obstetric outcomes and fetal well-being. According to several large studies on pregnancy outcomes among women complicated with asthma [1,2,3,4,5], most of them indicate that pregnancies complicated with asthma are associated with an increased risk for various adverse obstetric outcomes, such as preeclampsia, gestational diabetes mellitus, cesarean section rate, preterm birth, low birth weight, and intrauterine growth restriction. The association was still significant even after controlling for potential confounding factors. However, the results are relatively conflicting. Some studies indicated that women with asthma had comparable reproductive risks compared with those without asthma in the general population for most obstetric outcomes [6]. Although several studies have reported on this issue, the conclusions are still conflicting. In addition, in the large studies, the data were based on computerized primary care databases or registered databases without comprehensive review and validation of the data directly from the medical records, resulting in less reliable and less informative data for analysis. Most importantly, the patients with asthma in most previous studies were heterogeneous in terms of severity, in spite of the fact that intermittent and persistent asthma of various levels of severity may theoretically result in different outcomes. Accordingly, though many studies show that asthma during pregnancy poses a common, increasingly prevalent threat to the health of mothers and their babies, more reliable studies on this issue are still required. However, asthma usually induces systemic inflammatory chemicals, associated with poor pregnancy outcomes, especially preterm birth or preeclampsia. We hypothesized that such poor outcomes might be associated only with the severe cases, and it may be not appropriate to use this information for counseling all pregnant patients with asthma. We wonder whether intermittent asthma, the least severe form but accounting for the majority of cases, increases such adverse outcomes or not. This information is clinically important and may contribute to the antenatal management of these patients which account for the largest group of asthma during pregnancy. Therefore, we conducted this study aimed to compare the adverse pregnancy outcomes between low-risk pregnancy complicated with intermittent asthma and low risk pregnancies without any risk factor for adverse pregnancy outcomes. Additionally, we aim to perform subgroup analysis to compare such outcomes between the controls and cases of intermittent asthma with active disease during pregnancies.

## 2. Materials and Methods

A retrospective cohort study, using a prospective database, was carried out at a university hospital, a tertiary care referral center (Maharaj Nakorn Chiang Mai Hospital, Thailand). The prospective database was developed as a part of fellowship (subspecialty) training in maternal–fetal medicine (MFM). On the database creation, all consecutive cases of pregnant women diagnosed with asthma were reviewed at the time of discharge after birth and prospectively recorded for demographic data, natural course of the disease, management, and pregnancy outcomes. This study was conducted with ethical approval of the Institutional Review Board, Faculty of Medicine, Chiang Mai University; study code: OBG 2562-06967. The study period included the time range between 2004 and 2020. On research conduct, the database was accessed to retrieve the digital records of singleton pregnancies diagnosed with asthma. Moreover, the medical records of the study cases were comprehensively reviewed and validated by the authors. The study group (asthma) included women meeting the following criteria: (1) singleton gestation with reliable gestational age according to fetal ultrasound in the first half of pregnancy, (2) documented diagnosis of intermittent asthma (defined, in case of no treatment, as symptoms of ≤2 day/week, nocturnal awakenings ≤ 2 episodes/month, no interference with normal activity, and normal lung function between exacerbations [1]) and treated by our internists (Pulmonary Unit, Department of Internal Medicine, Faculty of Medicine), most treated with beta-agonists and inhaled corticosteroid, (3) attending antenatal care clinic and having delivery at our hospital, (4) no other coincidental medical disorders, e.g., chronic hypertension, thyrotoxicosis, cardiac disease etc., (5) smoking or drug abuse, (6) known outcomes of the pregnancy. The women in the control group were recruited from our database of low-risk pregnancies during the same study period. The records of controls were randomly retrieved, with a control-to-case ratio of 10:1. The controls were also validated by the authors, based on the same inclusion criteria used for the study group but had no asthma. The exclusion criteria for the two groups were as follows: fetal structural or chromosomal abnormalities, pregnancies complicated with other underlying diseases, diagnosis of persistent asthma, and incomplete data of the pregnancy outcomes.

The full medical records of the patients in the study group (asthma) were thoroughly reviewed and validated for baseline characteristics, details of the clinical course of the disease (e.g., severity of asthma, activity of the disease, medications used, time and number of asthmatic attacks, predisposing factors such as smoking, time point of first diagnosis) and laboratory reports. The women in the study group were sub-divided into 2 subgroups: active and inactive disease during pregnancy. The activity was simply defined as active disease if there was at least one asthmatic attack per pregnancy course, in spite of previously under control with proper treatment, based on documentation of the internists who took care of the patients.

All of the recruited women in both groups were comprehensively reviewed for the common adverse pregnancy outcomes as follows: abortion (miscarriage at or prior to 20 weeks of pregnancy), stillbirth (birth of the baby with no Apgar score), preterm birth (birth prior to 37 complete weeks of pregnancy), intrauterine growth restriction (birth weight less than 10th percentile of the standard growth chart), low birth weight (birth weight of less than 2500 g), preeclampsia (a new onset of hypertension in the second half of pregnancy together with proteinuria which was defined as 24-h urine protein of greater than 300 mg), gestational diabetes mellitus based on the two-step criteria recommended by American College of Obstetricians and Gynecologists (ACOG), antepartum hemorrhage, route of delivery (cesarean section rate), low Apgar scores (the score of less than 7 at 5 min), and postpartum hemorrhage. The pregnancy outcomes mentioned above were compared between (1) the study groups and the control groups, and (2) the control group and the subgroup of patients with active asthma during pregnancy.

Statistical analysis: All of the data was analyzed using the statistical package for the social sciences (SPSS) software version 26.0 (IBM Corp. Released 2019. IBM SPSS Statistics for Windows, Version 26.0 Armonk, New York, NY, USA). Regarding the baseline characteristics, continuous data were presented as mean+ SD or median (IQR), as appropriate, whereas the categorical data were presented as percentage. In the comparison of the adverse outcomes between the two groups, Chi-square test as well as relative risk with 95% CI was used for categorical variables, and *t*-tests were used for the continuous variables. A *p*-value of less than 0.05 was defined as having statistical significance.

## 3. Results

**Figure 1:**
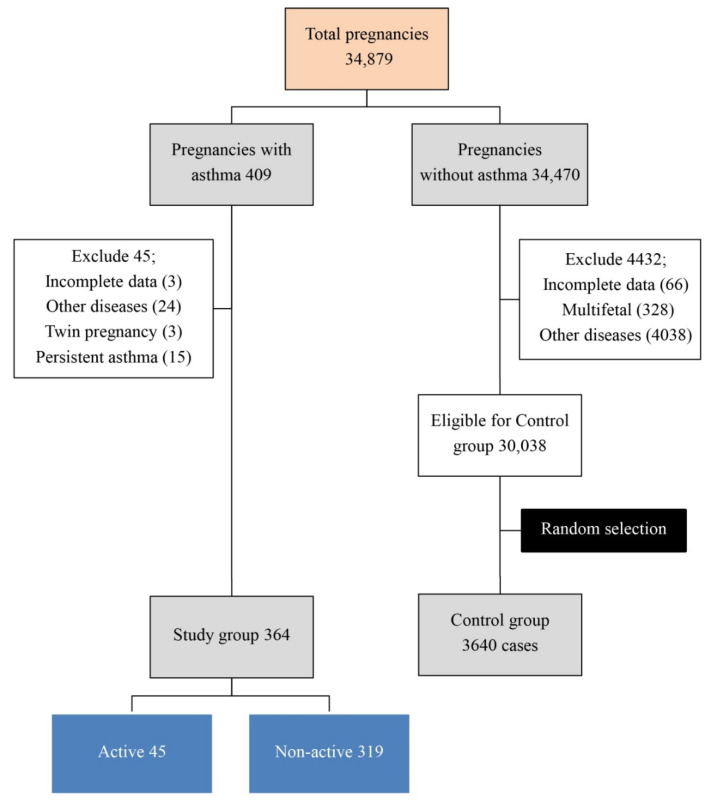
Flowchart of patient recruitment.

During the study period, the prevalence of pregnant women complicated with asthma was found to be approximately 1.2% of total birth (409 out of 34,879 pregnancies). About 3.7% (15 out of 409 cases) were classified as persistent asthma, whereas most of them (96.3%) were classified as intermittent asthma. Of 409 cases of maternal asthma, 45 were excluded from analysis because of several reasons as presented in Figure 1. 

The remaining 364 cases with intermittent asthma and 3640 cases of the control group were available for analysis. Demographic characteristics of the women in the study group and the control group were not significantly different, as presented d in Table 1. 

Among the patients in the study group, 45 cases were classified as active cases whereas the remainders were not active. Of the 45 cases, most (31 cases) had one episode of asthmatic attack, 12 had two episodes, and two had three episodes. Of 61 episodes, 70.5% (43 attacks) occurred in the second trimester, while the occurrence in the first and third trimester was five and 13 episodes, respectively.

### 3.1. Comparison between the Study Group and the Control Group

The rates of the adverse pregnancy outcomes in the patients with intermittent asthma and the women in the control group were not significantly different in all of the outcomes, as presented in Table 2. 

Moreover, the mean ± SD gestational age at delivery and the mean ± SD birth weight in the two groups were not significantly different (*p*-value: 0.704 and 0.797, respectively). It is noteworthy to observe that the patients with intermittent asthma had a trend of a higher rate of fetal growth restriction (13.3% vs. 6.6%), though the difference was not statistically significant.

### 3.2. Comparison between the Group of Active Cases and the Control Group

Interestingly, subgroup analysis comparing the adverse outcomes between women with active intermittent asthma and the controls showed that the mean ± SD gestational age at delivery and the mean ± SD birth weight in the groups of active disease were significantly lower than those in the control group (*p* < 0.05), as presented in Table 3. 

Notably, the rates of fetal growth restriction, preeclampsia, and preterm birth tended to be higher in the group of active asthma, though the difference did not reach the statistically significant level. Interestingly, the rates of fetuses with low-birth weight and low Apgar scores were significantly higher in the cases with active intermittent asthma (*p* < 0.05). Likewise, in the study group (patients with intermittent asthma), when comparing the obstetric outcomes in the active subgroup with those in the non-active subgroup, the results are similar to the comparison between the active subgroup and normal controls, as presented in Table 3.

## 4. Discussion

A new insight gained from this study is that intermittent asthma is not associated with poor pregnancy outcomes, but cases with active disease may slightly increase risk of preterm birth and low birth weight. Though this study seems to be a negative report, the findings can have clinical impact. As previously mentioned, asthma seems to pose many risks to the mother and fetus, such as preeclampsia, gestational diabetes, preterm birth, low birth weight, fetal growth restriction, and an increased cesarean section rate, but our results suggest that those poor outcomes may be associated only with the severe cases, and it may be not appropriate to use such information for counseling all patients with asthma. Preferably, the counseling should follow tailored management. The obstetric outcomes among women with intermittent asthma are more favorable than ever thought. Actually, if we exclude the active cases from analysis, the rates of common adverse outcomes were very similar to those in the control group.

The conflicting data on the outcomes presented in the previous studies [1,4,6] might partly be explained by different proportions of the severe cases. In most large national database, severity of asthma might not be digitally entered and unavailable for analysis and impossible to perform subgroup analysis. Based on our finding and most previous studies, an increased risk of poor pregnancy outcomes might be confined only in the group of persistent asthma or the more severe cases.

Asthma is an inflammatory disease of the respiratory tract which can release a variety of inflammatory factors to implicate placental function and development [7,8], possibly leading to an increased risk of poor obstetric outcomes, including pre-eclampsia, preterm birth and fetal growth restriction. Several studies demonstrated that asthma significantly increases the risk of preeclampsia, possibly as high as 50%. Nevertheless, this may be confined in the group of symptomatic asthma during pregnancy [9] or had history of admissions or emergency visits prior to the current pregnancies [10,11], reflective of severity dependence. This is also in agreement with the study reported by Schaltz et al. [12], who found that preeclampsia was significantly associated with lower FEV1, indicating that the severity of asthma must play an important role. Martel et al. [11] supported that relationship by demonstrating that inhaled SABA use to control the severity during pregnancy could reduce the risk of preeclampsia. Again, our study reaffirmed that intermittent asthma, the least form of severity, did not increase the risk of preeclampsia, either active or not.

Many studies have linked maternal asthma to increased incidence of gestational diabetes mellitus, possibly as many as 40% [5,13]. According to both retrospective and prospective studies, maternal asthma was an independent factor for risk of gestational diabetes, after adjustment for confounding factors, and was minimized by active treatment. The pathophysiology is unclear, but it is possible that maternal BMI, known risk factor of gestational diabetes, can be a potential confounder, since obesity is common in women with asthma. Moreover, this may be induced by oral corticosteroids, commonly used in severe or active asthma, as suggested by one previous study [14]. Again, the evidence suggests that the increased risk of gestational diabetes tends to be confined in the cases of more severe. This may explain the non-increase in our study that included only intermittent asthma.

Note that the rate of preterm birth in the cases of active asthma tended to increase but did not reach statistical significance, while the mean gestational age was significantly lower. The two findings were in agreement to support an increase in preterm birth rate. Accordingly, it is convincing that with more sample size the subtle significance of the difference will be more obviously expressed. The prevalence of low birth weight was significantly increased in cases of active disease. Interestingly, the rate of fetuses with low Apgar scores at 5 min was slightly but significantly higher in the active group. The findings may be reflective of overall adverse fetal outcomes, likely caused by preterm birth or low birth weight. It is unclear how asthma can increase preterm birth or low birth weight, but it is possibly associated with inflammatory factors, increased in asthmatic patients, which is already known that they can initiate labor.

In most previous studies, cesarean rate was reported to be higher among pregnancies with asthma. Nevertheless, the increased cesarean rate can be expected to be higher, going along with increased rates of poor obstetric outcomes. As seen in general practice, the cesarean rate is relatively high among complicated pregnancies, e.g., preeclampsia, diabetes mellitus, and fetal growth restriction. However, our finding indicated that cesarean rate was not increased in cases of intermittent asthma, when compared with the controls. Certainly, severity of the disease could explain this discrepancy. Likewise, Wang et al. [5] demonstrated that the risk of cesarean section is higher for patients with severe asthma when compared to mild asthma.

The strengths of this study are as follows: (1) Although this is a retrospective cohort study, the data were obtained by comprehensive review and validation of the full medical records, not just derived from crude databases without details for validation. (2) Homogeneous data of the cases with intermittent asthma contributes to a more reliable conclusion. (3) Common, known confounding factors that might possibly affect the primary outcomes were excluded. Limitations of the study include: (1) The sample size is probably too small to address the outcomes with low occurrence rate like stillbirth or abortion and also too small for subgroup analysis. (2) The nature of the study was retrospective. (3) The study did not cover cases of asthma with more severity or persistence (mild, moderate, severe).

Clinical impact: Our findings may have implications for prenatal care of the pregnant women with intermittent asthma. We can counsel the couples regarding the more favorable outcomes in pregnancy with intermittent asthma, but caution should still be exercised in taking care of the active cases since poor outcomes can be minimized by effective care or prevention of the exacerbations [15]. Probably, active disease or exacerbations are the key events that may contribute to poor obstetric outcomes.

## 5. Conclusions

Low-risk pregnant women with intermittent asthma generally have incidences of adverse pregnancy outcomes similar to those in low-risk pregnancies without asthma. The patients with intermittent asthma have better pregnancy outcomes than ever reported in patients with mixed groups of severity. Nevertheless, though the disease is classified as intermittent asthma, if the disease is active during pregnancy, with at least one attack during pregnancy, the pregnancies tend to carry a higher risk of preterm birth and low birth weight fetuses, but further studies are needed to confirm this. The insights gained from this study are useful to support counseling and management of patients with intermittent asthma, which is the majority of asthmatic patients during pregnancy.

## Figures and Tables

**Table 1:** Baseline characteristics of pregnancies in the study group (pregnancies with asthma) and the control group.

Outcome	Study Group (*n* = 364)	Control Group (*n* = 3640)	*P*-Value
Mean maternal age; Year ± SD	28.2 ± 6.1	28.3 ± 6.0	0.800
Number of antenatal care visits: No. ± SD	9.1 ± 8.8	8.8 ± 3.7	0.159
Private practice:			0.280
• Private patients	42 (11.6%)	493 (13.6%)	
• General patients	321 (88.4%)	3133 (86.4%)	
Parity:			0.209
• Nulliparous	212 (58.2%)	1995 (54.8%)	
• Parous women	152 (41.8%)	1645 (45.2%)	
Residency:			0.067
• Chiang Mai	270 (74.2%)	2532 (69.6%)	
• Others	94 (25.8%)	1108 (30.4%)	

**Table 2:** Comparisons of the pregnancy outcomes between pregnancies with asthma vs. controls.

Outcomes	Case *N* = 364	Control *N* = 3640	Relative Risk (95% CI)	*P* Value
Quantitative outcomes	Mean ± SD	Mean ± SD		
Gestational weeks	37.2 ± 3.8	37.1 ± 4.5	-	0.704
Birth weight (grams)	2847 ± 685	2837 ± 775	-	0.797
Categorical outcomes	n/N (%)	n/N (%)		
Abortion	6/364 (1.6%)	99/3640 (2.7%)	0.6 (0.3–1.4)	0.223
Antepartum hemorrhage	3/364 (0.8%)	45/3640 (1.2%)	0.7 (0.2–2.1)	0.491
Gestational diabetes	35/364 (9.6%)	354/3640 (9.7%)	0.8 (0.3–2.2)	0.946
Preeclampsia	24/364 (6.6%)	195/3640 (5.4%)	1.2 (0.8–1.9)	0.323
Cesarean delivery	70/364 (19.2%)	849/3640 (23.3%)	0.8 (0.7–1.1)	0.077
Postpartum hemorrhage	1/364 (0.3%)	17/3640 (0.5%)	0.6 (0.1–4.4)	0.601
Preterm birth < 37 wk	55/364 (15.1%)	558/3640 (15.3%)	1.0 (0.8–1.3)	0.912
Fetal growth restriction	6/364 (13.3%)	242/3640 (6.6%)	2.0 (0.9–4.3)	0.075
Low birth weight	65/364 (17.9%)	636/3640 (17.5%)	1.0 (0.8–1.3)	0.854
Low Apgar (5 min)	21/364 (5.8%)	224/3640 (6.2%)	0.9 (0.6–1.4)	0.770
Stillbirth	7/364 (1.9%)	118/3640 (3.2%)	0.6 (0.3–1.3)	0.168

**Table 3:** Comparisons of the pregnancy outcomes between pregnancies with active cases in the group of intermittent asthma vs. controls and active asthma vs. non-active asthma (within the group of intermittent asthma).

Outcomes	Active Cases *N* = 45	Control *N* = 3640	*P* Value *	Non-Active Cases *N* = 319	*P* Value **
Quantitative outcomes	Mean ± SD	Mean ± SD		Mean ± SD	
Gestational weeks	35.3 ± 4.2	37.1 ± 4.5	0.007	37.5 ± 3.7	0.001
Birth weight (grams)	2517 ± 819	2837 ± 775	0.006	2894 ± 652	0.001
Categorical outcomes	n/N	n/N		n/N	
	(%)	(%)		(%)	
Abortion	1/45	99/3640	0.838	5/319	0.747
	(2.2%)	(2.7%)		(1.6%)	
Antepartum hemorrhage	1/45	45/3640	0.554	2/317	0.268
	(2.2%)	(1.2%)		(0.6%)	
Gestational diabetes	5/45	354/3640	0.755	30/319	0.716
	(11.1%)	(9.7%)		(9.4%)	
Preeclampsia	5/45	195/3640	0.090	19/319	0.192
	(11.1%)	(5.4%)		(6.0%)	
Cesarean delivery	11/45 (24.4%)	849/3640 (23.3%)	0.860	59/319 (18.5%)	0.343
Postpartum hemorrhage	1/45	17/3640	0.093	0/319	0.124
	(2.2%)	(0.5%)		(0.0%)	
Preterm birth < 37 wk	11/45	558/3640	0.093	44/319	0.062
	(24.4%)	(15.3%)		(13.8%)	
Fetal growth restriction	6/45	242/3640	0.075	20/319	0.085
	(13.3%)	(6.6%)		(6.3%)	
Low birth weight	13/45	636/3640	0.046	52/319	0.039
	(28.9%)	(17.5%)		(16.3%)	
Low Apgar (5 min)	6/45	224/3640	0.048	15/319	0.020
	(13.3%)	(6.2%)		(4.7%)	
Stillbirth	1/45	118/3640	0.701	6/319	0.876
	(2.2%)	(3.2%)		(1.9%)	

* Comparing between active cases of Study group and Control group. ** Comparing between active cases and non-active cases in Study group.

## Data Availability

The datasets analyzed during the current study are available from the corresponding author upon reasonable request.
